# Biomechanics of bone-fracture fixation by stiffness-graded plates in comparison with stainless-steel plates

**DOI:** 10.1186/1475-925X-4-46

**Published:** 2005-07-27

**Authors:** VK Ganesh, K Ramakrishna, Dhanjoo N Ghista

**Affiliations:** 1 iDAF Systems; Block 2019, # 03-252; Bukit Batok Street 23; 659524, Singapore; 2 School of Mechanical and Aerospace Engineering; Nanyang Technological University; 50 Nanyang Avenue; 639798, Singapore; 3 Division of Bioengineering; Nanyang Technological University; 50 Nanyang Avenue; 639798, Singapore

## Abstract

**Background:**

In the internal fixation of fractured bone by means of bone-plates fastened to the bone on its tensile surface, an on-going concern has been the excessive stress-shielding of the bone by the excessively-stiff stainless-steel plate. The compressive stress-shielding at the fracture-interface immediately after fracture-fixation delays callus formation and bone healing. Likewise, the tensile stress-shielding of the layer of the bone underneath the plate can cause osteoporosis and decrease in tensile strength of this layer.

**Method:**

In order to address this problem, we propose to use stiffness-graded plates. Accordingly, we have computed (by finite-element analysis) the stress distribution in the fractured bone fixed by composite plates, whose stiffness is graded both longitudinally and transversely.

**Results:**

It can be seen that the stiffness-graded composite-plates cause less stress-shielding (as an example: at 50% of the healing stage, stress at the fracture interface is compressive in nature i.e. 0.002 GPa for stainless steel plate whereas stiffness graded plates provides tensile stress of 0.002 GPa. This means that stiffness graded plate is allowing the 50% healed bone to participate in loadings). Stiffness-graded plates are more flexible, and hence permit more bending of the fractured bone. This results in higher compressive stresses induced at the fractured faces accelerate bone-healing. On the other hand, away from the fracture interface the reduced stiffness and elastic modulus of the plate causes the neutral axis of the composite structure to be lowered into the bone resulting in the higher tensile stress in the bone-layer underneath the plate, wherein is conducive to the bone preserving its tensile strength.

**Conclusion:**

Stiffness graded plates (with in-built variable stiffness) are deemed to offer less stress-shielding to the bone, providing higher compressive stress at the fractured interface (to induce accelerated healing) as well as higher tensile stress in the intact portion of the bone (to prevent bone remodeling and osteoporosis).

## Background

Fracture-fixation by bone-plate is intended to provide immobilization at the fracture site and reduce the fracture gap, thus allowing primary bone-healing or healing by endosteal callus formation (for micro-movement in order of 500 microns). The role of bone-plate and screws is to hold the fractured bone segments in position, without allowing tensile stresses at the fractured interface but rather have some critical compressive stress induced in it so as to accelerate healing. The complications associated with plate fixation are loosening of screws under loading, local effects on vascularity of the cortex beneath the plate (blocking normal blood flow), and (from a biomechanics viewpoint) excessive shielding of stresses from the bone [1-3].

The biomechanics factors, governing the healing efficiency in fractured bone treated by plate and screws, are: (1) the degree of bone contact developed at the fracture interface, (2) stability provided to the fractured bone in terms of reduced movement at the fracture interface, and (3) necessary and sufficient stress-shielding of the bone at fracture interface as well as away from it. Hitherto, conventional high-stiffness stainless-steel (SS) have been employed for long-bone fracture-fixation. However, the big difference in modulus between the plate and bone as well as the compressive stresses occurring between the plate and the bone (due to over-tightening of screws) disturb the vascularity of the bone underneath the plate, causes bone resorbtion underneath the plate and reduction in its strength as a long term effect.

In recent years, there has been considerable awareness and discussion on the need for using less-stiff plates to improve fracture healing and prevent bone weakening due to stress-shielding [2-10]. It is not entirely correct to say that bone-plates with high stiffness (or Young's modulus 'E') cause excessive stress-shielding, because stiffness is characterized by the product E and moment of inertia of the plate cross-section; hence the plate geometry also has a bearing on the stiffness as thereby on the stress-shielding of the bone. However, for a uniform plate geometry, plates with a lower E will offer less stress shielding than the plates with higher Young's modulus [11].

### Materials involved in bone-plate design

The biocompatible materials used for bone plates are: stainless steel (SS), cobalt base alloys, bioceramics, titanium alloys, pure titanium, composite materials, and polymers (non-resorbable and bioresorbable). Each of the above materials can broadly be categorized as (i) bioinert (ii) porous, (iii) bioactive, and (iv) bioresorbale [12]. In general, bioinert material is selected for bone-plates because bioactive material gets bonded with the bone (along with the soft tissues) and causes problems if plate removal or corrective surgery is required.

The bioceramic materials which are bioinert (like Al_2_O_3_, ZrO_2_), possess Young's modulus (E) in the range of 400 ± 20 GPa, in contrast to that of hydroxyapatite. While the properties of ceramics (such as high hardness, chemical inertness, oxidation resistance, high strength, high melting points and low fracture toughness) are suited to the requirement for the bone-plate, its brittleness and high 'E' result in stress-shielding of the bone, thus limiting its use for bone-plates [13].

Metallic alloys like Cobalt-base alloys (e.g CoCrW, CoCrMo) have 'E' of about 250 ± 10 GPa along with wear, corrosion and heat resistances. However, they are not suitable for usage, owing to their poor fabricability and high cost [14]. Stainless steel (e.g 316L) is one of the most preferred biomaterials for bone-plates, because of its mechanical properties ('E = 200 ± 20 GPa', ductility etc), corrosion resistance, bioinert and cost-effectiveness in comparison with other biocompatible metals [15]. Titanium alloys (e.g Ti-6Al-7Nb, Ti-6Al-4V), with E of 110 ± 10 GPa, are especially preferred for bone screws, because of their increased corrosion resistance and improved ductility. However, although titanium alloys offer improved strength (with less ductility) compared to pure titanium, they are not preferred for plate implants because of difficulty in their contouring (as required for pelvic and mandibular plates). Titanium alloys are however preferred for intramedullary rods, spinal clamps, self-drilling bone screws and other implants, because of their high strength and low 'E' [16].

Pure Titanium metal is also one of the most widely chosen materials for the bone-plates, because of its excellent biocompatibility and corrosion resistance. The ductility of titanium is less compared to SS, because of its hexagonal crystal structure. This makes contouring of titanium plates difficult, compared to stainless steel plates. Titanium plates also offer less stress-shielding to bone (for the same geometries) after healing, because its 'E' is 68 GPa compared to 200 GPa of SS [17]. However, they are not as amenable to contouring as SS plates.

Composite materials (e.g. Carbon Fiber Reinforced Polymers, CFRP) which consist of a polymer matrix and fibre, which are combined to achieve the requisite high strength and adequate 'E' value. The polymer matrix materials can be broadly classified as resorbable (e.g. polysorb, biosyn) and nonresorbable (such as PEEK, ultrahigh molecular weight polyethylene or UHMWPE). Polymers per se do not have the strength and stiffness required for bone-plates; hence polymers reinforced by fibers are employed for the bone-plate application or used as scaffolds in the preparation of bone grafts [18]. Composite materials used for bone-plates mainly consist of a thermoplastic polymer matrix (such as polyetheretherketone or PEEK, polymethylmethacrytale or PMMA etc.) and fibres such as glass or carbon. The disadvantage of using composite material arises is that in case of implant failure, when revision surgery is warranted. This is because of the risk of fibre breakage and subsequent penetration of small fibre particles into the bone tissue, causing irritation and inflammation [19].

The increased use of bioresorbable polymers (i.e. polymers which degrade in-vivo to non-harmful by-products) in the recent year's poses the problem of their strength loss while bone-healing is in progress [20]. It is to be noted that bone-plate fracture-fixation should sustain loads for 1.5 to 2 years [21], which is yet to be achieved with resorbable materials. Hence, a new class of resorbable materials needs to be developed, having adequate mechanical properties and resorbtion time increased by 1 to 2 years.

In view of the above discussion, polymers and calcium phosphates are osteoinductive and resorbable; they cannot behave as load-sharing members and fail in in-vivo loading conditions [22]. For a reinforced fractured bone, it is important to initially have a plate with sufficient stiffness so as to prevent tensile stresses at the fracture interface, while allowing the bone away from the fracture site to be stressed under loading conditions (so as to prevent loss of bone strength). An optimal plate needs to be designed such that it caters to the above mentioned objectives.

Based on these considerations, we recommend the use of stiffness-graded materials (SGMs) for bone-plates. SGMs are characterized by a smooth and continuous change of the mechanical properties from one characteristic surface to the other. Stiffness-graded material is a relatively new concept in bone-plates in order to decrease stress shielding (this concept is well documented for dental implants) [23-26]. Controlled segregation, controlled blending, vapor deposition, plasma spraying, electrophoretic deposition, controlled powder mixing, slipcasting, sedimentation forming, centrifugal forming, laser cladding, metal infiltration, controlled volatilization, and self propagating high-temperature synthesis are few manufacturing techniques that are involved in fabrication of SGMs. Current production of SGMs is hampered by the current manufacturing process technology.

In this paper, a preliminary comparison of the stiffness graded plates with stainless steel plates is provided, with respect to bone healing stages and stress-shielding by means of finite element analysis. Herein, we have explored the viability of using stiffness-graded materials as bone-plates, in order to reduce the stress-shielding effect, by providing an inside view of the stresses in bone during various stages of healing.

Axial compressive load is more prominent in long bones [27]. However, it does not endanger bone-healing by opening the fracture gap and it contributes to more interfragmentary compression at fracture interface. On the other hand, load eccentricity from the center of the bone-plate and the intrinsic curvature of long bones cause bending moments to be applied to the fracture fixed bone. Bending moment will induce both tension and compression stresses across the fracture interface, and open up the fracture, leading to the reduction in the stability of the fixation. Hence, bending loading is considered by us for finite element analysis of plate-reinforced bone.

## Finite element method (for analysis of plate-reinforced fractured bone)

### Finite element model

**Figure 1:**
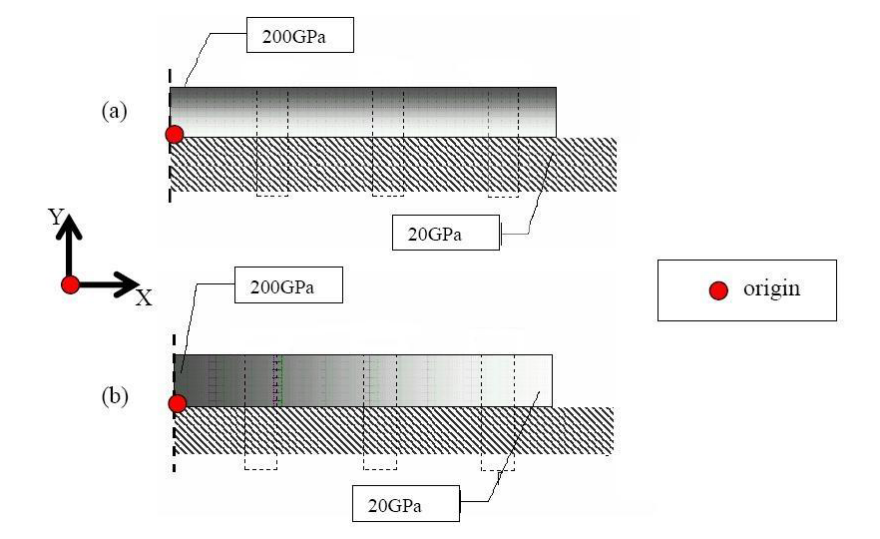
Fracture fixation plates (length 60 mm and thickness 5 mm) with grading (a) Stiffness graded along thickness and is given by Young's modulus = 36X+20 (b)Stiffness graded along length Young's modulus = -6X+200.

A two-dimensional model of plate-reinforced fractured bone is analyzed, using ANSYS (commercially available finite element code). The geometry of the long bone (tibia) is obtained through a digitizer (outer diameter of bone at mid cortex is 15 mm and the cortical thickness is 4.5 mm), and is imported into ANSYS for analysis. The bone material is assumed to be isotropic and uniform throughout the bone, with a Young's modulus of 20 GPa and Poisson-ratio 0.3. While long bone (eg. tibia) are transversely isotropic, we have adopted isotropy herein for convenience sake, because our objective is to demonstrate the effect of graded plates on reduction of bone stress shielding. Herein, three different plates (of length 60 mm and thickness 5 mm) are considered for analysis: (i) Stainless-steel plate, with a uniform E of 200 GPa throughout the length of the plate, (ii) Stiffness-graded plate along the thickness (SGT), wherein the E of the plate is 200 GPa at the top layer of the plate and decreases linearly towards the bottom of the plate to 20 GPa and (iii) Stiffness-graded plate along the length (SGL), having E of 200 GPa in the middle section of the plate and decreasing linearly towards the end of the plate to 20 GPa (shown in figure 1).

From a structural analysis consideration, the plate-bone assembly is analyzed as a composite beam (plate is fixed onto the bone). A unit bending moment of 1 Nmm is applied on the fracture-fixed bone. A transverse fracture (i.e. fracture gap) of 1 mm thickness is incorporated into the model. Callus is assumed to bridge the fracture gap. The callus material is assumed to be isotropic and homogeneous, having E = 0.02 GPa at 1% healing (at initial stages of healing i.e. 1^st ^week of healing), 10 GPa at 50% healing (3^rd ^week of healing), 15 GPa at 75% healing (final stages of healing before remodeling i.e. at 6^th ^week of healing) [28,29].

## Analysis and results

### (a) For a stainless-steel plate fixation

**Figure 2:**
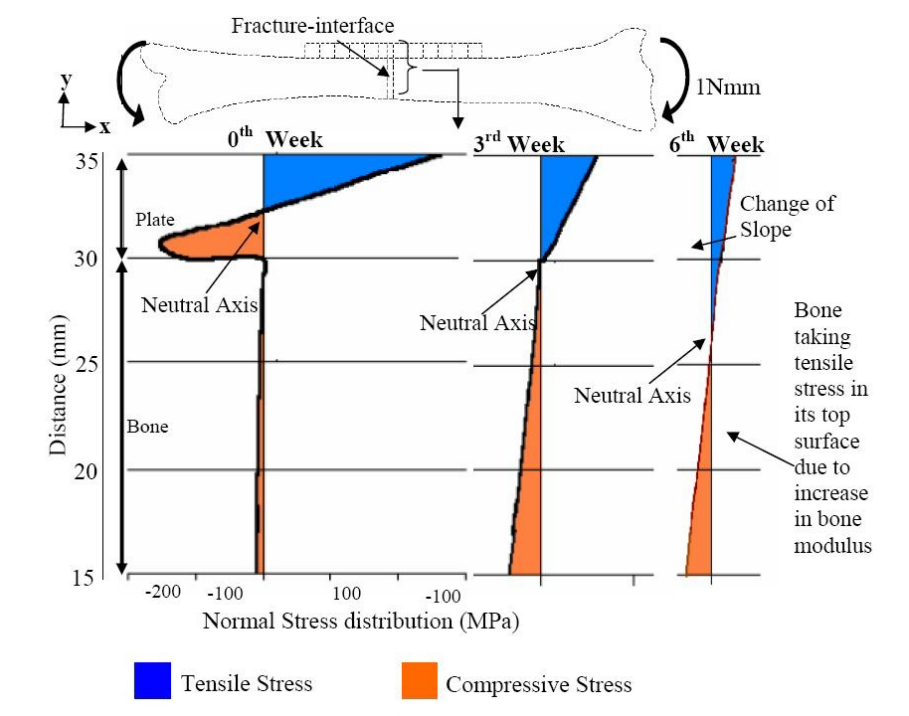
Normal stress S_xx _(MPa) at the fracture interface for different stages of healing with SS plate.

Figure 2 illustrates how the stress at the fracture-interface varies with time, due to fracture healing. The healing is simulated by adopting callus E 0.02 GPa at 1% healing, 10 GPa at 50% healing and 15 GPa at 75% healing. Initially (at 1% healing), the neutral axis is located in the middle of the plate, because the loading bearing cross-section at the fracture-interface consists only of the plate. Three weeks later (at 50% healing), as some callus develops at the fractured interface, the neutral axis shifts into the bone-plate interface. Hence, the callus bone is able to take on some compressive stress.

Six weeks later (at 75% healing), because there is more callus consolidation, the modulus of the laid-over bone at the fractured interface increases considerably, and the neutral axis shifts into the bone domain. The maximal tensile stress in the plate decreases, while the maximal compressive stress in the bone increases. Even after complete healing of the bone (i.e. at 100% healing), the plate will behave as a load-sharing member away from the fracture interface, and will reduce the stresses in the bone according to the composite beam theory.

Hence, in order to optimize the fracture-healing process, so as to enable the bone to start taking on stress early-on, it is desirable to have fixation plates with stiffness graded along the length and thickness (SGT and SGL) as illustrated by figure 1.

### (b): Stress variation at the bone fracture-interface, due to the SGT and SGL plate fixations at different stages of bone-healing

**Figure 3:**
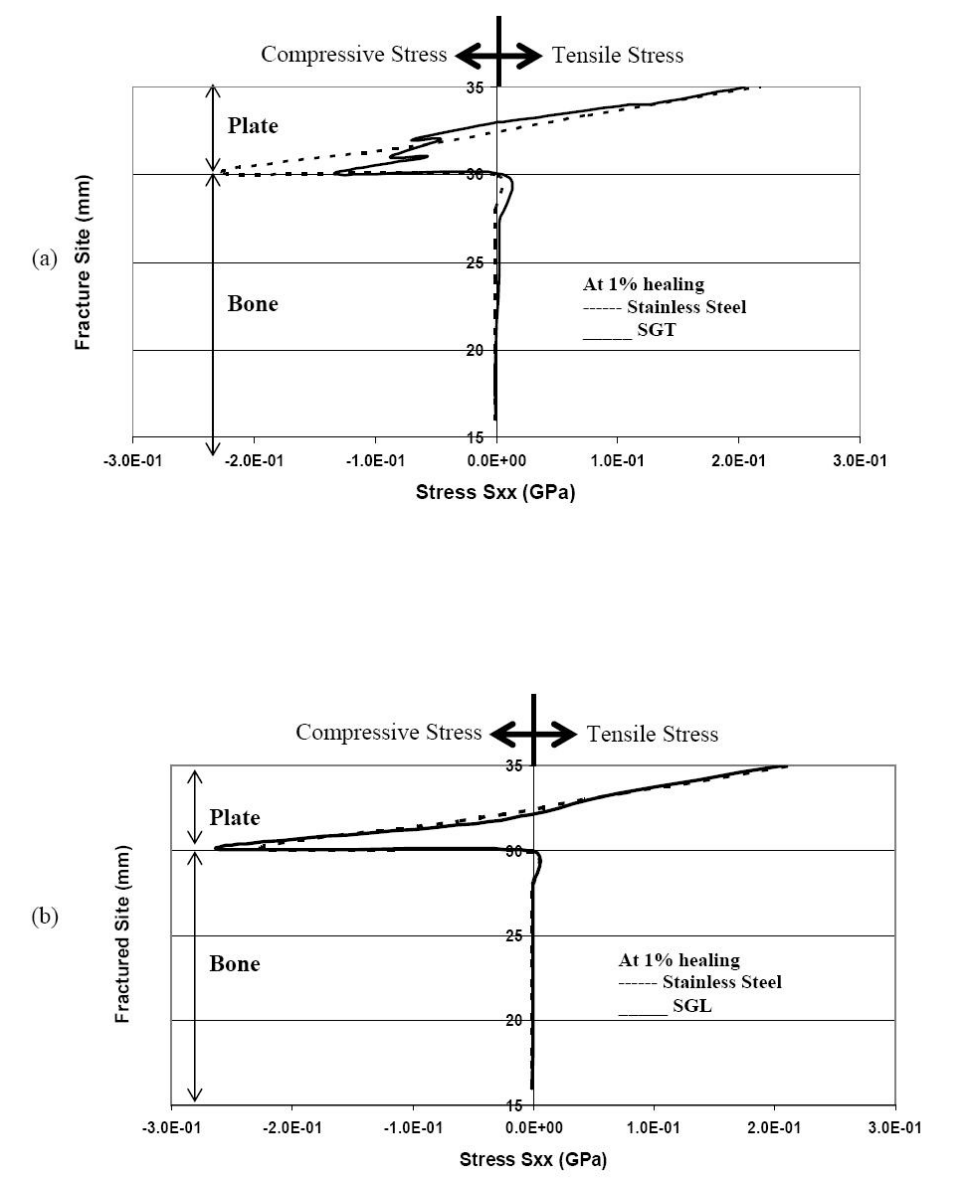
Normal stress S_xx _(GPa) at the fracture interface during 1% healing (a) comparison of stresses for SGT and SS (b) comparison of stresses for SGL and SS.

Figures 3a &3b illustrate that at 1% healing (when there is hardly any callus formed), the neutral axis is located inside the plate domain for all the three types (SS, SGT, SGL) of plate-fixation. So, even these graded plates protect the fractured bone by not allowing any tensile stress in the upper bone layers, and thereby provide a conducive healing environment. However, even these two graded plates do not provide sufficient compressive stress in the callus. This is not conducive to further callus formation, because some critical amount of compressive stress is needed to stimulate callus formation. Compression in the callus can be better achieved by pre-tensing or prebending the plate or through the application of the lag screw across the fracture. Perhaps, geometry modification in the plate design (by incorporating a spring-like effect within it) could bring about compression at the fracture interface.

**Figure 4:**
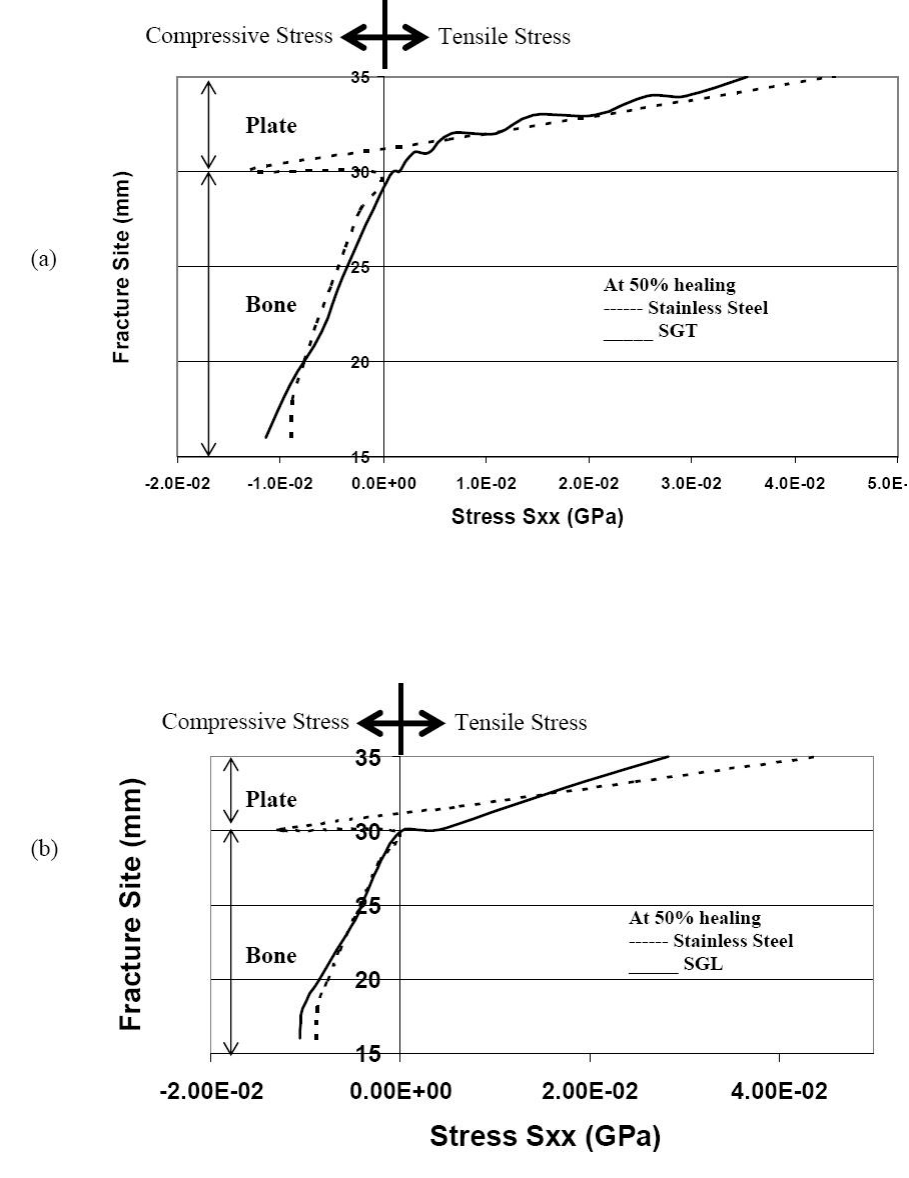
Normal stress distribution at the fracture interface (during 50% healing) (a) comparison of stresses for SGT and SS (b) comparison of stresses for SGL and SS.

As bone healing progresses to 50% callus formation, the neutral axis shifts down to the bone-plate interface for the SGT plates. Figures 4a &4b represent the status at 50% healing. In the case of SGL plate (and the SS plate), the neutral axis (NA) is still located inside the plate, and continues to stress-shield the bone. However, in the case of the SGT plate, the NA moves a bit into the bone (~1 mm from bone and plate interface). Hence, the SGT plate stress-shields the bone a little less than the SGL plate, and allows tensile stress in the bone layer underneath the plate.

**Figure 5:**
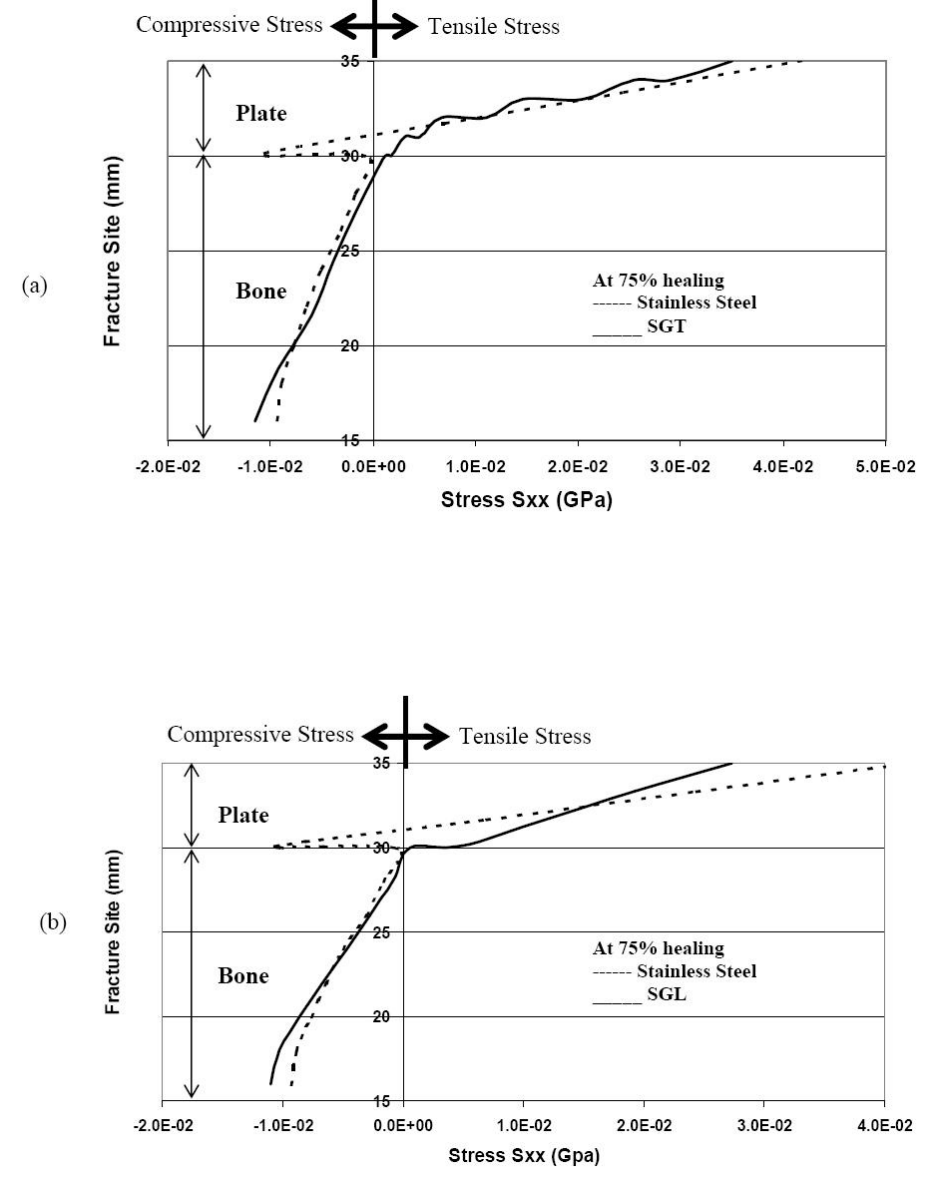
Normal stress distribution at the fracture interface during 75% healing stage (a) comparison of stresses for SGT and SS (b) comparison of stresses for SGL and SS.

Now even as healing proceeds to 75% full-healing and more bone is laid down, in the SS plate-fixation, the neutral axis is still within the plate domain (as seen in figure 5a &5b). Hence, the SS plate continues to shield the bone from acquiring any tensile stress and hence the bone will not be remodeled for sustaining tensile stress. In other words, the fractured faces are still entirely compressed. On the other hand, for the SGT and SGL plate fixations, the NA has moved a bit into the bone (for SGT ~2 mm and SGL ~1 mm from bone and plate interface). Both the SGT and SGL allow the bone to have tensile stress, which is conducive to remodeling of the bone. Compared to the SGL, the SGT allows the upper bone layer to be subjected to more tensile stress (1.2 times). As healing progresses, the SGT and SGL plates appear to be transmitting more compressive stress to the lower faces of the bone compared to the stainless steel plates.

### (c): Stress-distribution along the top and bottom layers of the fractured bone, due to the SS, SGT and SGL plate fixations, at different stages of bone-healing

**Figure 6:**
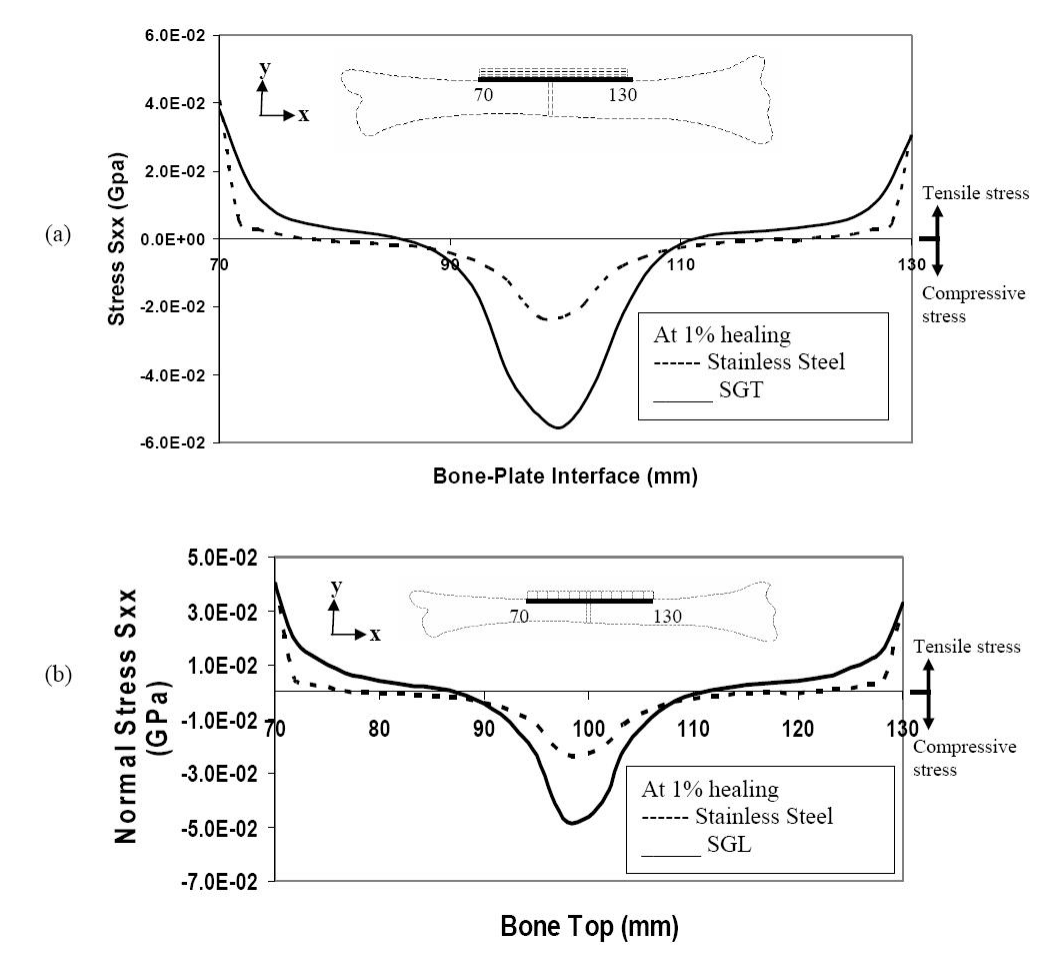
Stresses along the bone-plate interface (top layers of bone) during the initial stages of healing (a) comparison of stresses for SGT and SS (b) comparison of stresses for SGL and SS.

We will first study the stresses in the top layer of the bone. Right after fracture (1% healing), all the three types of plates shield the bone, by not allowing any tensile stress in the upper layers (underneath the plate) close to the fracture-interface. In other words, at cross-sections close to the fracture site, the neutral axis is located inside the plate. However, after about 10 mm from the fracture site, the neutral axis gets lowered into the bone region. Hence, bone cross-sections away from the fracture-site get subjected to tensile stress. It is seen that the SS plate allows less compressive stress in the bone surface close to the fracture and less tensile stress away from the fracture, compared to the SGT and SGL plates (figure 6a &6b). Based on the results depicted in the figure, the SGT plate may be regarded as more beneficial for accelerating callus formation and bone healing compared to SGL plate, because it provides more compression at the fracture interface and more tensile stress on the bone-plate interface away from fracture interface. The order of providing favorable-to-normal conditions for healing is SGT, SGL and SS.

**Figure 7:**
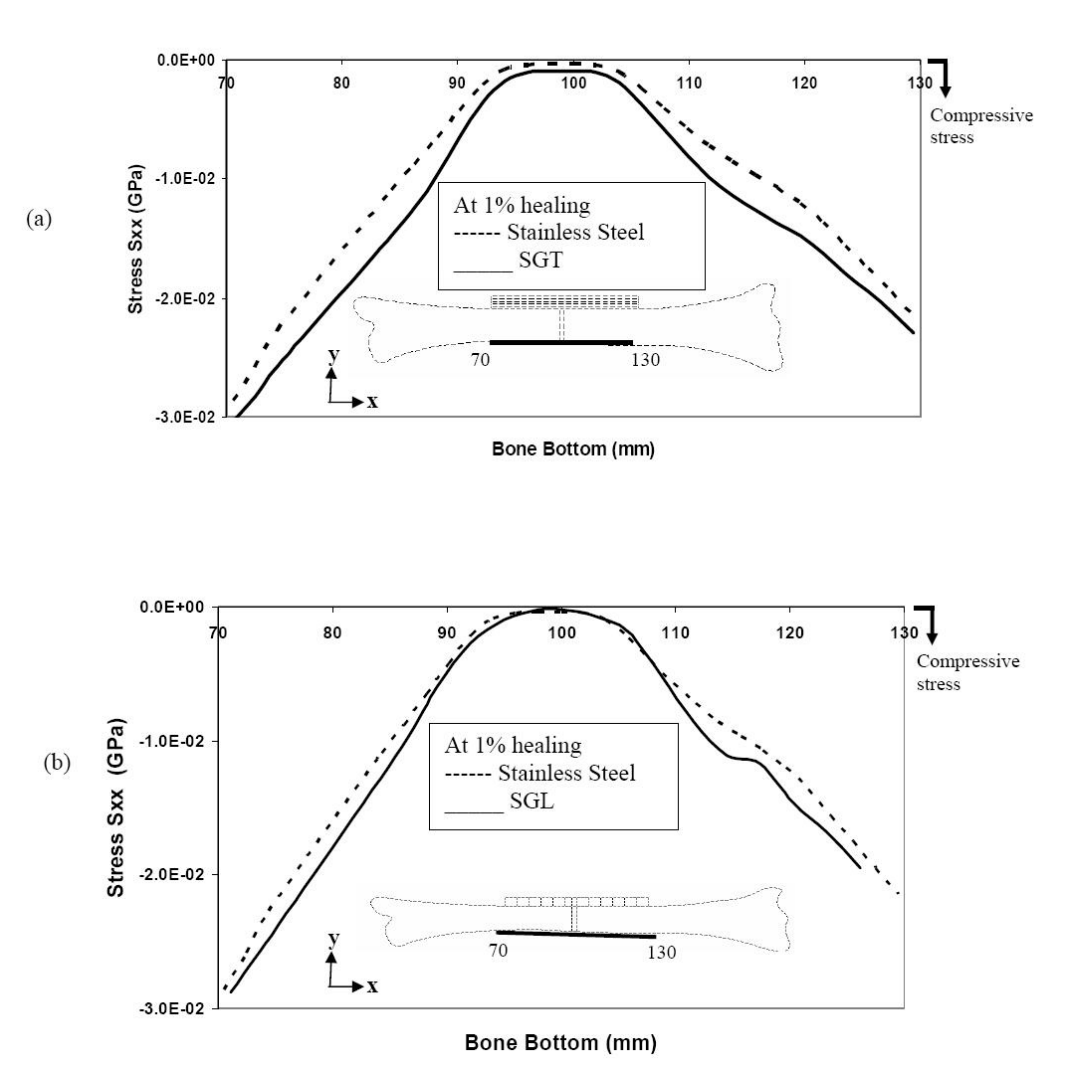
Stresses at the bottom layers of bone during the initial stages of healing (a) comparison of stresses for SGT and SS (b) comparison of stresses for SGL and SS.

Now, let us see what is happening in the bottom layer of the bone. Immediately after fracture-fixation (1% healing), the SGT, SGL and SS plates totally stress-shield the bone from any tensile stress along the bottom surface of bone (figure 7a &7b). On the other hand, the SGT allows slightly more compression along the bottom surface of the bone compared to SGL and SS (1.1 and 1.3 times respectively).

**Figure 8:**
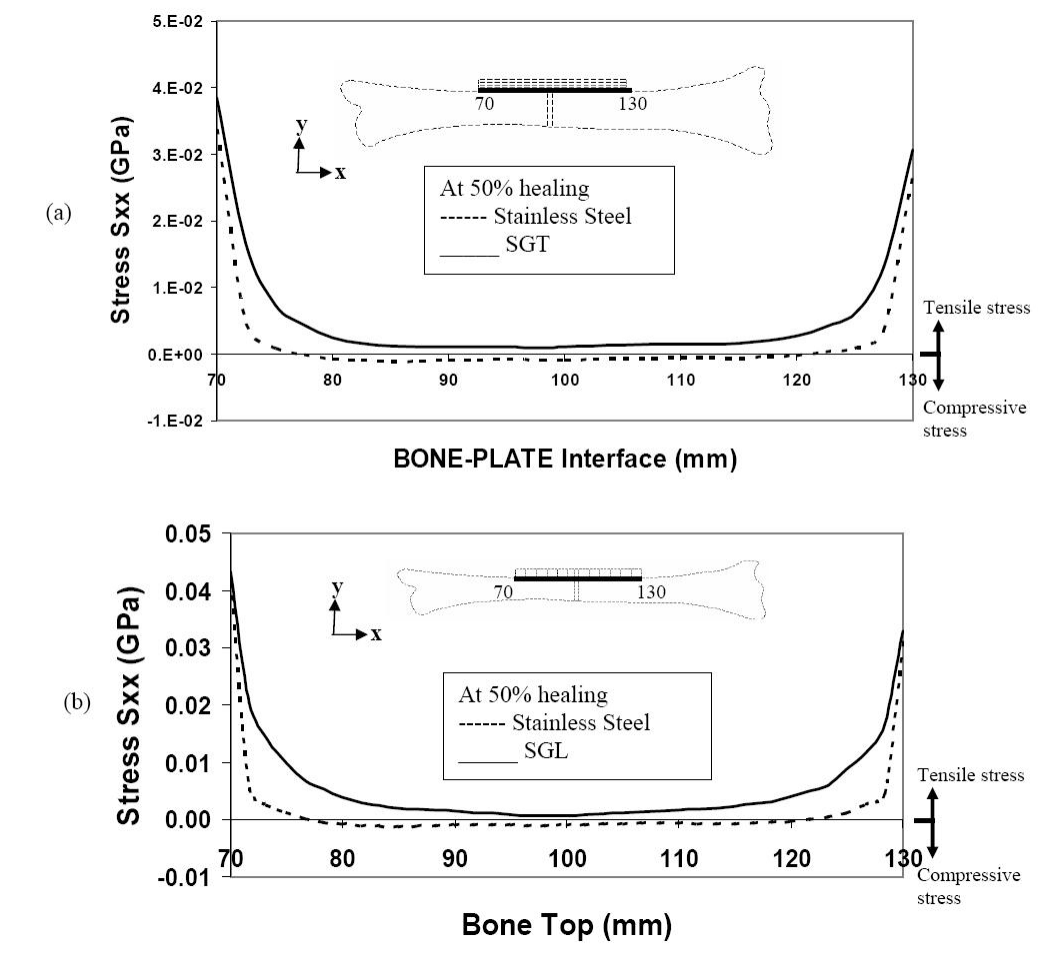
Stresses along the bone-plate interface during 50% healing (a) comparison of stresses for SGT and SS (b) comparison of stresses for SGL and SS.

Even at 50% bone-healing, the SS plate contributes to shield the bone by not allowing tensile stress in the bone layers underneath the plate. On the other hand, the SGT and SGL plates are allowing these bone layers to be subjected to some tensile stress (figure 8a &8b).

**Figure 9:**
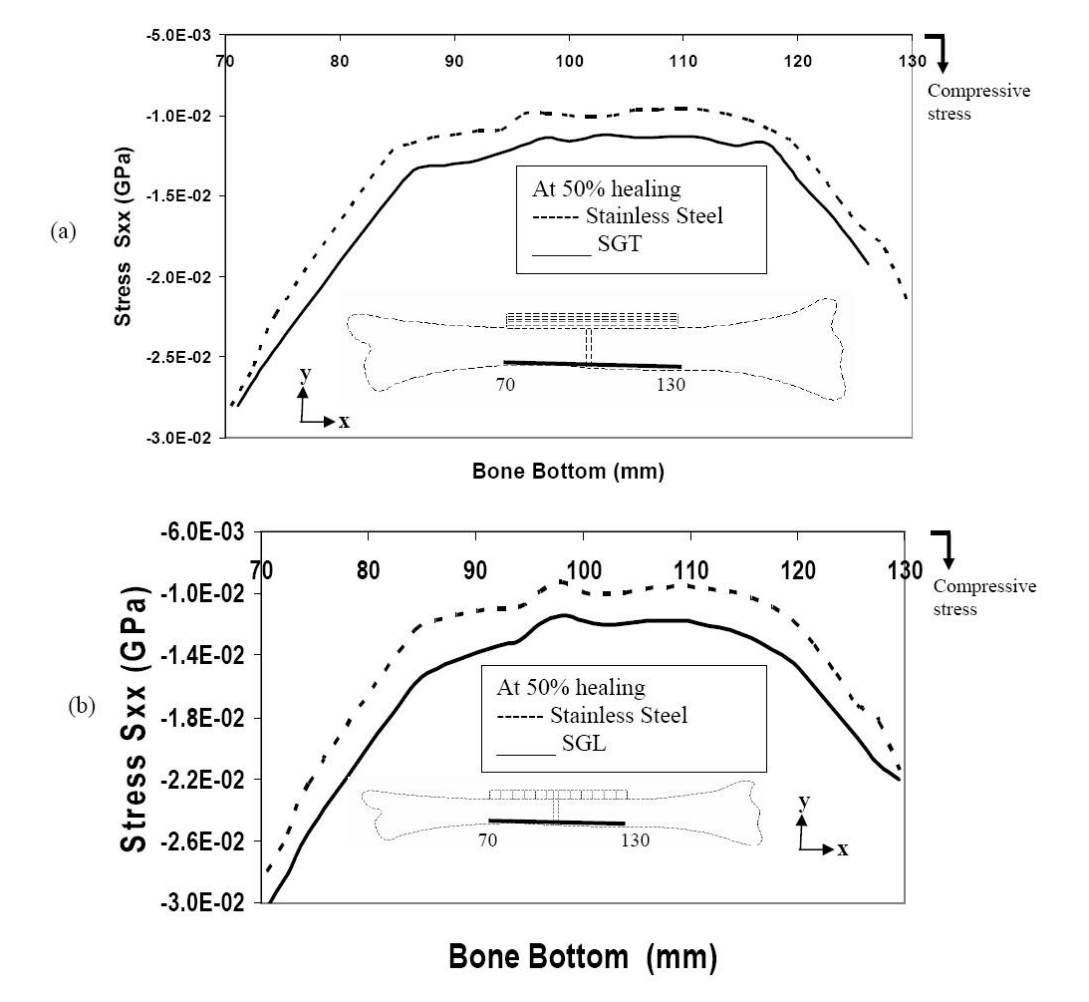
Stresses along the bottom of the bone during 50% healing (a) comparison of stresses for SGT and SS (b) comparison of stresses for SGL and SS.

At this stage of bone healing (50% healing), compressive stress is incurred in the bottom layers of the bone, for all (SGT, SGL and SS) types of plate-fixation. The longitudinal stress-distribution in the bottom layer of the bone for SGT plate is quite similar to that for the SGL plate (figure 9a &9b).

**Figure 10:**
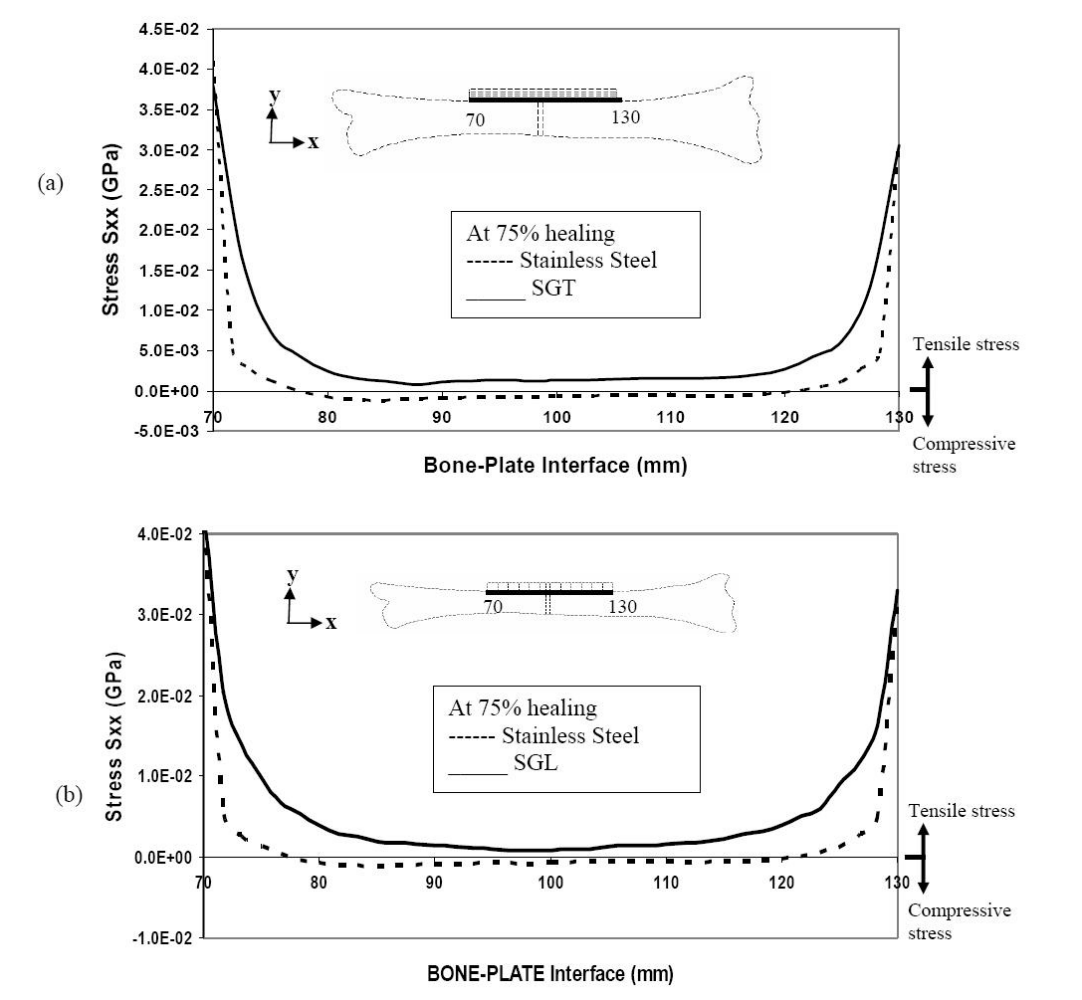
Stresses along the bone-plate interface during the final stages of healing (a) comparison of stresses for SGT and SS (b) comparison of stresses for SGL and SS.

**Figure 11:**
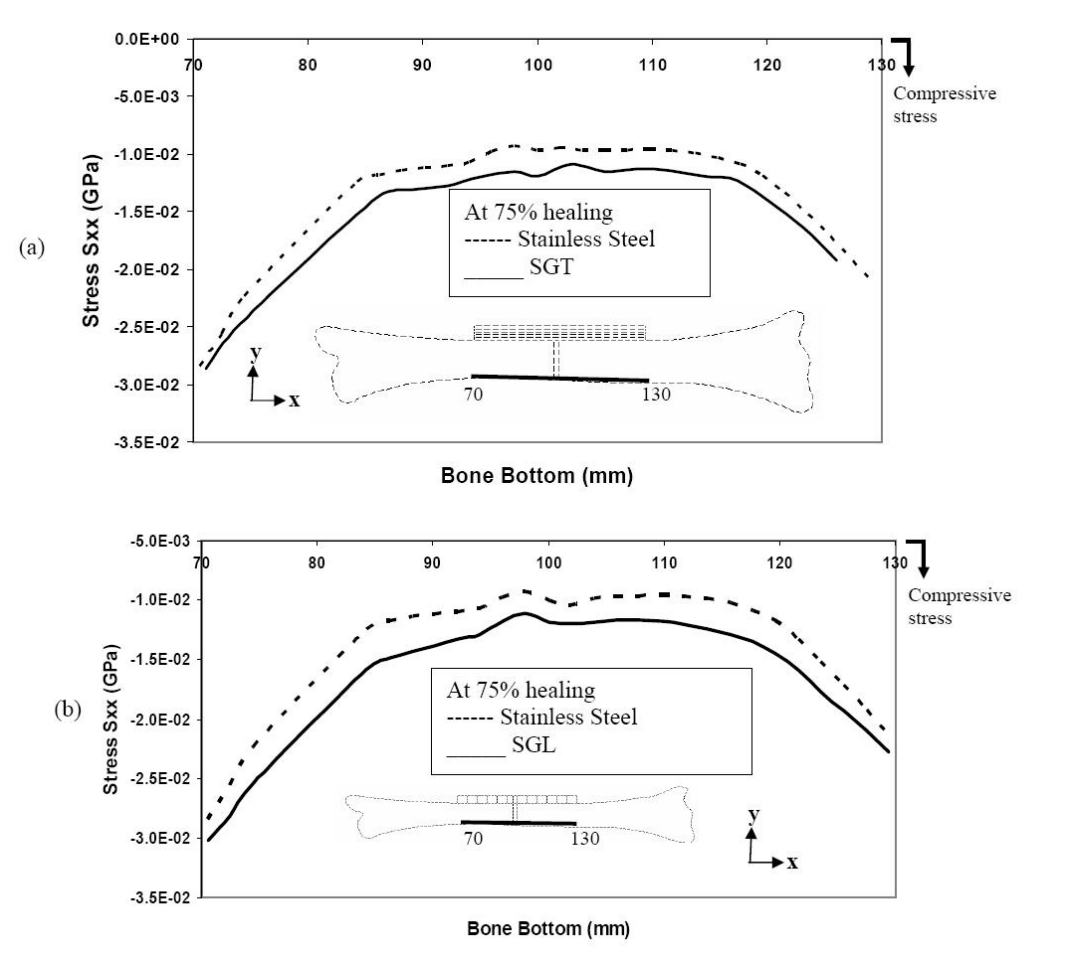
Stresses along the bottom layer of the bone during the final stages of healing (a) comparison of stresses for SGT and SS (b) comparison of stresses for SGL and SS.

Even at 75% bone healing, the SS plate still does not allow any tensile stress in the top layer, at and close to the fracture site. However, the SGT plate and the SGL plate allow the bone to experience similar levels of tensile stress, both close and away from fracture-site (figure 10a &10b). Even in an advanced stage of healing, the SS plate allows less compressive stress in the bottom layer of the bone compared to the SGT and SGL plates (figure 11a &11b).

## Conclusion

For the purpose of effectively conveying the information that we have been discussing earlier, we have represented the 50% healing results for all the three types of fixation in table-form. From table 1, it is evident that stiffness graded plates (both SGT and SGL) provide less stress shielding. At the fracture site, the SS plate continues to shield the bone (at its top layer, i.e at the bone plate interface) from tensile stress, whereas SGT and SGL allow the bone to take on some tensile stresses. Once the callus is mature (say at 50% healing), tensile stress at this stage of healing is beneficial for enhanced callus formation. Away from the fracture site, SGT and SGL allow the bone to take on more tensile stress compared to the SS plate, thus allowing the bone to retain its tensile strength properties.

## Authors' contributions

Dr. Ganesh did the analysis and Ramakrishna (Doctoral Student) prepared the paper under the supervision of Professor Dhanjoo Ghista.

**Table 1:** Comparison of stresses (at fracture site and 20 mm away from the fracture) during 50% bone healing for SS, SGT and SGL. Compressive stress is represented by a negative sign, while tensile stress is indicated by a positive sign.

	Top layer of the bone		Bottom layer of the bone	
	
Plate type	Stresses (10^-2 ^GPa) at fracture site	Stresses (10^-2 ^GPa) at 20 mm away from fracture site	Stresses (10^-2 ^GPa) at fracture site	Stresses (10^-2 ^GPa) at 20 mm away from fracture site
SS	-0.2	-0.02	-1	-1.5
SGT	+0.2	+0.4	-1.3	-1.9
SGL	+0.1	+0.6	-1.2	-2.1
